# Establishment of Quantitative Severity Evaluation Model for Spinal Cord Injury by Metabolomic Fingerprinting

**DOI:** 10.1371/journal.pone.0093736

**Published:** 2014-04-11

**Authors:** Jin Peng, Jun Zeng, Bin Cai, Hao Yang, Mitchell Jay Cohen, Wei Chen, Ming-Wei Sun, Charles Damien Lu, Hua Jiang

**Affiliations:** 1 Program for Computational Biology, Systems Biology, and Translational Research, Metabolomics and Multidisciplinary Laboratory for Trauma Research, Institute for Disaster and Emergency Medicine, Sichuan Provincial People's Hospital, Sichuan Academy of Medical Sciences, Chengdu, Sichuan Province, China; 2 Department of Trauma Surgery, Sichuan Provincial People's Hospital, Sichuan Academy of Medical Sciences, Chengdu, Sichuan Province, China; 3 Department of Computational Mathematics and Biostatistics, Metabolomics and Multidisciplinary Laboratory for Trauma Research, Institute for Disaster and Emergency Medicine, Sichuan Provincial People's Hospital, Sichuan Academy of Medical Sciences, Chengdu, Sichuan Province, China; 4 Department of Surgery, San Francisco General Hospital, University of California San Francisco, San Francisco, California, United States of America; 5 Department of Parenteral and Enteral Nutrition, Peking Union Medical College Hospital, Beijing, China; Hertie Institute for Clinical Brain Research, University of Tuebingen, Germany

## Abstract

Spinal cord injury (SCI) is a devastating event with a limited hope for recovery and represents an enormous public health issue. It is crucial to understand the disturbances in the metabolic network after SCI to identify injury mechanisms and opportunities for treatment intervention. Through plasma 1H-nuclear magnetic resonance (NMR) screening, we identified 15 metabolites that made up an “Eigen-metabolome” capable of distinguishing rats with severe SCI from healthy control rats. Forty enzymes regulated these 15 metabolites in the metabolic network. We also found that 16 metabolites regulated by 130 enzymes in the metabolic network impacted neurobehavioral recovery. Using the Eigen-metabolome, we established a linear discrimination model to cluster rats with severe and mild SCI and control rats into separate groups and identify the interactive relationships between metabolic biomarkers in the global metabolic network. We identified 10 clusters in the global metabolic network and defined them as distinct metabolic disturbance domains of SCI. Metabolic paths such as retinal, glycerophospholipid, arachidonic acid metabolism; NAD–NADPH conversion process, tyrosine metabolism, and cadaverine and putrescine metabolism were included. In summary, we presented a novel interdisciplinary method that integrates metabolomics and global metabolic network analysis to visualize metabolic network disturbances after SCI. Our study demonstrated the systems biological study paradigm that integration of 1H-NMR, metabolomics, and global metabolic network analysis is useful to visualize complex metabolic disturbances after severe SCI. Furthermore, our findings may provide a new quantitative injury severity evaluation model for clinical use.

## Introduction

Spinal cord injury (SCI) is a major public health challenge that often affects young adults. In the United States, 12,000 new SCI cases are annually reported, and $24 billion is annually spent on paralyzed SCI patients [1]. The incidence of SCI in China is surging because of the large population and rapid economic growth; in Beijing the incidence has reached 60/10^6^ per year [2]. SCI is a complex injury that involves multiple pathological processes, particularly when the injury is severe, and triggers shock and organ dysfunction or death. Understanding the cellular and metabolic network malfunction during SCI is crucial for clinical monitoring and intervention.

Human metabolism is a complex network with hundreds of cross-linked paths. During critical SCI illness, the metabolic network is dynamically disturbed at multiple points [3]. Classical research typically isolates a small part of this network to investigate the impact of pathophysiological molecular mechanisms on clinical outcome. In particular, researchers have examined metabolic disturbances such as skeletal muscle breakdown, insulin resistance, dyslipidemia, testosterone and growth hormone/IGF-I dysfunctions, low thyroxine syndrome, and deficiency of vitamin D and calcium with secondary hyperparathyroidism [3]–[14]. These complex metabolic disturbances appear and interact at different stages during the pathological process after SCI [3], [4]. Therefore, an integrated approach that combines the biochemical/molecular changes with network disturbances is the key to understanding SCI at the systems biology level and establishing an accurate quantitative model for monitoring SCI.

An interdisciplinary method that includes high-throughput quantitative techniques and effective mathematical and visualization tools is necessary because metabolic network disturbances after SCI are complex; hundreds of molecules and metabolic paths interact [15], [16]. Furthermore, interdisciplinary methods present the opportunity to develop innovative clinical diagnosis and monitoring methods for severe injuries. Here we present a novel high-throughput method that integrated 1H-nuclear magnetic resonance (NMR) metabolomic fingerprinting with global metabolic network analysis to formulate a quantitative SCI evaluation model.

## Materials and Methods

### 1. Modeling Spinal Cord Injury in SD Rats

Animal modeling procedures were performed according to an established protocol, as described previously [17]. In brief, adult male Sprague–Dawley (SD) rats that had a normal neurobehavioral function were included in the study. The Basso, Beattie, and Bresnahan (BBB) score scale was used to evaluate neurobehavioral function (BBB scores >20 were considered normal, BBB scores of enrolled animals see Supporting Information: table S1 and table S3). Rats were randomly divided into three groups: control (*n* = 7), mild SCI (*n* = 10), and severe SCI (*n* = 25). All the rats were fed the standard formula feed from 7 days before the operation until sacrifice. All rat forage was formulated according to the formulation established by the State Bureau of Quality Technical Supervision of China (GB Standards No: 14924.3-2010). Animal use was strictly in accordance with the National Institutes of Health Guide for the Care and Use of Laboratory Animals. The Experimental Animal Committee of Sichuan Academy of Medical Sciences approved the research protocol (Approval Number: 10-1069).

Rats assigned to the SCI group were anesthetized by intra-peritoneal injection of sodium pentobarbital (40 mg/kg). We tried our best to minimize animal suffering. Rats were observed for at least 3 days after the start of the experiment. For severe SCI, we established a 7-day observation subgroup. In the mild SCI group, three rats died during the surgical modeling procedure and one died 2 days after the surgery. In the severe SCI group, five rats died during the surgical modeling procedure and four died 1 and 2 days after the surgery. Therefore, there were six animals in the mild SCI group, 16 in the severe injury group (seven rats were fed for 7 days after modeling), and seven in the blank control group. Plasma samples were collected for 1H-NMR metabolomic analysis.

### 2. 1H-NMR Metabolomic Testing

Plasma samples were centrifuged at 10,000 rpm for 10 min to remove particulate matter. Following this, 100 µL of deuterium oxide (D2O) was added to 400 µL of the plasma samples in 5 mm Wilmad NMR tubes to lock the field frequency for the NMR spectrometer.

A high-solution Bruker AVIII-DRX600MHz NMR spectrometer (600.13 MHz 1H-NMR observation frequency; Bruker Biospin Rheinstetten, Germany) was used to obtain 1H-NMR spectra from the plasma samples. The probe temperature was 300 K. One 1D-NMR experiment was included. A standard protocol that included a one-dimensional pulse sequence and a Carr–Purcell–Meiboom–Gill (CPMG) pulse sequence was followed [34]. All samples were subjected to a 64 ms spin–spin relaxation delay and a 2-s water suppression irradiation during the relaxation delay. The spectral width was 20 ppm, and 64 transients were collected into 64,000 data points. The CPMG pulse sequence was used to filter broad resonances from protein and lipids and to visualize the latent biomarkers of smaller molecules.

### 3. Data processing and multivariate analysis

Principle component analysis (PCA) and partial least-square (PLS) regression were performed on 1H-NMR data to reduce data dimensionality and for multivariate analysis. We used Matlab R2012b for computing and mathematical modeling (The Mathworks, Inc.; Natwick, MA, US). The variable importance for the projection (VIP) value was used to screen metabolites. The Human Metabolome Database (HMDB) was used to identify key metabolites related to enzymes and upstream genes. Metabolites and their enzymes were mapped, and the disturbed metabolic paths were located on a global metabolic map of the Kyoto Encyclopedia of Genes and Genomes (KEGG).

A linear discriminant formula was established as a quantitative injury evaluation model. In this model, weighted PLS-DA values were used. The root mean square error (RMSE) was calculated to verify the validity of the equations according to injury severity.

Metabolic disturbances after severe SCI were established according to two steps: 1) key metabolites were mapped onto the KEGG metabolic network (KEGG metabolic pathways PATHWAY: map01100) and 2) the shortest path for each key metabolite (node) was calculated and outlined onto the metabolic network using the KEGG pathway API for the Matlab bioinformatics toolbox.

## Results

### 1. Effects of SCI on the plasma metabolome

**Figure 1:**
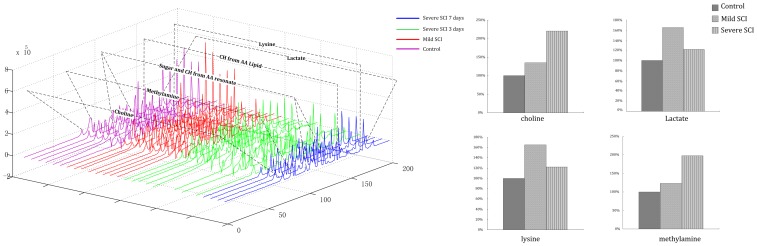
1H-NMR spectra. Purple lines represent the control group, red lines represent the mild injury group, green lines represent the severe injury group (3 days after injury), and blue lines represent the severe injury group (7 days after injury). Significant and distinguishable metabolomic changes occur after SCI. The right subplot shows the relative change in choline, lactate, lysine, and methylamine.

Compared with the control group, mild and severe SCI 1H-NMR spectra were perturbed at 0–8 ppm (Figure 1). This phenomenon suggests that the profiles of low-molecular-weight plasma metabolites and lipoproteins were altered by SCI. Visual inspection of the spectra demonstrated that there were significant alterations in plasma lactate, lysine, glucose, choline, and lipids (Table 1 and Figure 1).

**Table 1:** Summary of Eigen-metabolome: metabolites related to severe SCI.

Categories	Metabolite	Fold-change	*P*
Neural Injury Metabolism	4-Hydroxybutyric acid	1.2	ns
	Choline	0.7	<0.01
	L-Arginine	1.2	ns
	L-Histidine	1.0	ns
	L-Methionine	2.1	<0.01
	Tyramine	2.3	<0.01
	L-Serine	1.8	ns
Energy Flow	L-Lactic acid	2.4	<0.01
	Malonic acid	1.2	ns
Skeleton Muscle	Phosphocreatine	0.8	<0.01
	3-Indolebutyric acid	0.7	<0.01
	3-Methylhistamine	1.3	ns
	Putrescine	2.1	<0.01
Urine Impairment	Urea	2.1	<0.01
	Inosine	1.5	0.03

Abbreviation: ns = no significance.

**Figure 2:**
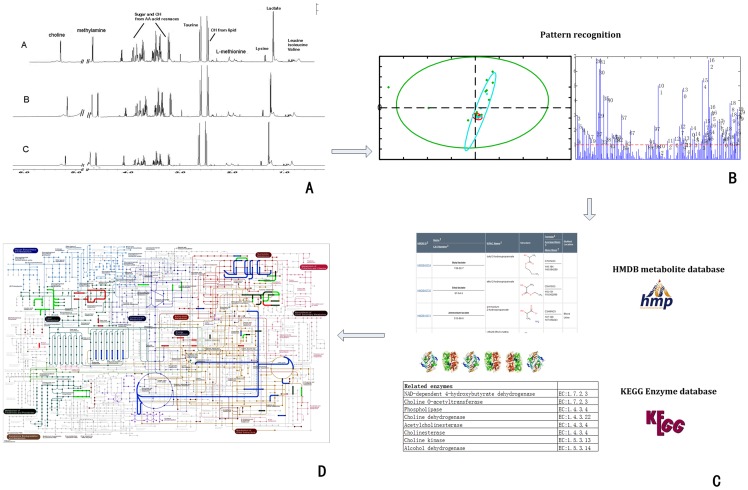
Metabolic disturbance visualization after SCI: study design. (A) 1H-NMR spectra (600 MHz) of plasma treated with a piecewise integral to form a 29×200 matrix. (B) PCA and PLS. Variable importance for the projection (VIP) values were used to screen metabolites. (C) The Human Metabolome Database (HMDB) was used to identify enzymes and genes that metabolize and regulate key metabolites. (D) Mapped metabolites and their enzymes at a global metabolic pathway in KEGG formed the visualization of metabolic disturbances after SCI.

- **PCA.** PCA scores indicated that injured rat plasma metabolomes differed from controls. The first two principal components (PCs) explained 83% of the total variance and suggested that most metabolome differences between normal and SCI cases were because of injury (Figure 2A).

### 2. The PLS model: severe SCI Eigen-metabolomes

PLS was used to investigate correlations between severity of SCI and the metabolome. The VIP indicator can describe correlations between the variable (X) and response (Y). We used VIP to identify metabolites correlated with severe SCI and named these metabolites as “Eigen-metabolome” of severe injury. Segmental integrations in 83, 90, 92, 151, 152, and 153 were found to have the highest VIP values and represented metabolites with 4.72, 4.44, 4.4, 2, 1.98, and 1.96 1H-NMR spectra chemical shift values, respectively.

We identified 15 metabolites that made up an “Eigen-metabolome” capable of distinguishing rats with severe SCI from healthy control rats. We also found that 16 metabolites impacted neurobehavioral recovery (Table 1 and 2).

**Table 2:** Summary of Eigen-metabolome: metabolites related to neurobehavioral recovery.

Categories	Metabolites	Fold-change	*P*
Tissue damage and Inflammation	Trimethylamine	1.6	<0.01
	3-Methyhistamine	1.3	ns
Glucose Metabolism	Galactonic acid	1.8	<0.01
	Gluconic acid	1.3	ns
	D-Xylulose	0.5	<0.01
Neurotransmitters	Theophylline	1.1	ns
	Dopamine	0.8	<0.01
Lipids	Heptadecanoic acid	1.3	0.0318
	Pentadecanoic acid	1.1	0.047
	Glyceraldehyde	2.1	<0.01
Amino Acids	Dimethyl glycine	0.9	ns
	L-Serine	1.1	ns
	L-Glutamic acid	1.2	0.05
	L-Histidine	1.0	ns
	Oxalacetic acid	0.9	ns
	5-Aminolevulinic	1.3	<0.01

Abbreviation: ns = no significance.

### 3. Quantitative severe SCI discriminate model

**Figure 3:**
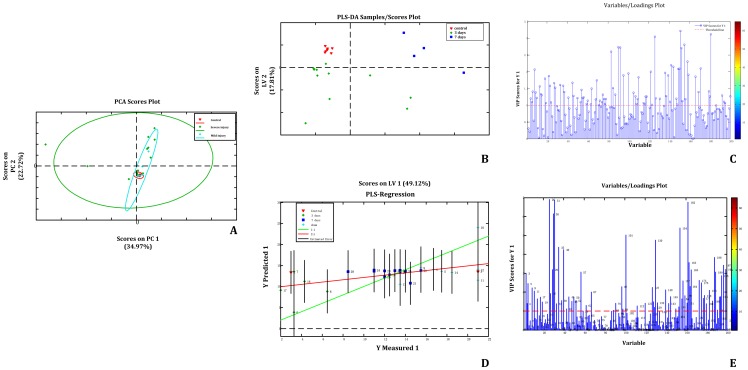
1H-NMR-based metabolomics spectrum: PCA and PLS. Pattern recognition of samples scored by PCA (A), PLS-DA (B and C), and PLS regression (D and E). (A) The PCA score plot of 1H-NMR spectra from plasma of controls (▾), severe injury (*), and mild injury (+). The ellipse was defined as the Mahalanobis distance from the distribution of samples. (B) Cross-validated scores plot of PLS-DA of the plasma 1H-NMR spectra for control (▾), mild injury (+), and severe injury (▪). (C) VIP plot of PLS-DA. The threshold line indicates VIP value >1. (D) Regression plot of PLS-R based on BBB scoring of animals before scarification; the red line indicates the best fit line, the straight green line represents a 1∶1 ratio, and the black line represents a 95% confidence interval. (E) Loading plot of PLS-R based on BBB scoring.

**Figure 4:**
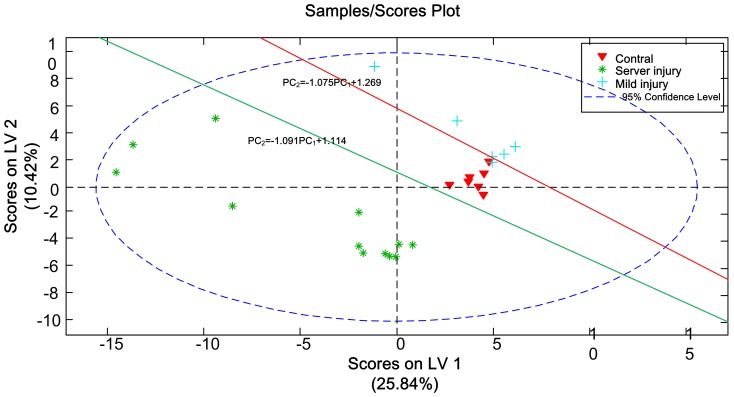
Severe and mild SCI score plot. The blue ellipse illustrates 95% CIs. The red and green lines represent SVM discriminant values. Multiclassification of severe injury (*), mild injury (+), and control samples (▴). The SVM linear discriminant surface is composed of the red and green lines. LV = latent variables.

To develop a quantitative severe SCI evaluation model based on the Eigen-metabolome, PLS regression analysis was performed to establish a linear prediction model (equation 1). The accuracy ratio of PLS regression was 86.2%, R^2^ = 0.284 (Figure 3F–G). *X_i_* represented the *i*-th ppm value from the H1-NMR spectra, where *i* = 1, 2, 3…, 200 (For raw data, see Supporting Information: table S2).
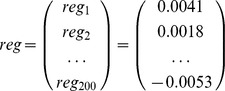
RMSE verified the validity of the PLS-regression model and was calculated according to the following formula:
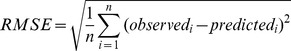
RMSE = 4.76 indicated that the model had an acceptable accuracy (for raw data, see Supporting Information: table S2.) Following this, the relationship between the discrimination equations was used to evaluate the relationship between plasma metabolites and injury severity, where *x* represented NMR spectra ppm values and *a_ij_* represented loadings.
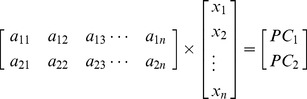
Finally, we arrived at an injury severity discrimination model based on the support vector machine (SVM).
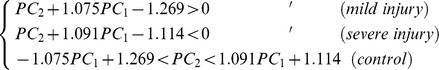
In summary, plasma NMR spectra could be clustered into one of three categories: control, mild injury, and severe injury. Only one mild injury sample was incorrectly clustered into the control category. All severely injured rats were separated from control and mildly injured rats (Figure 4).

### 4. Metabolic network disturbance visualization after SCI: KEGG mapping

**Figure 5:**
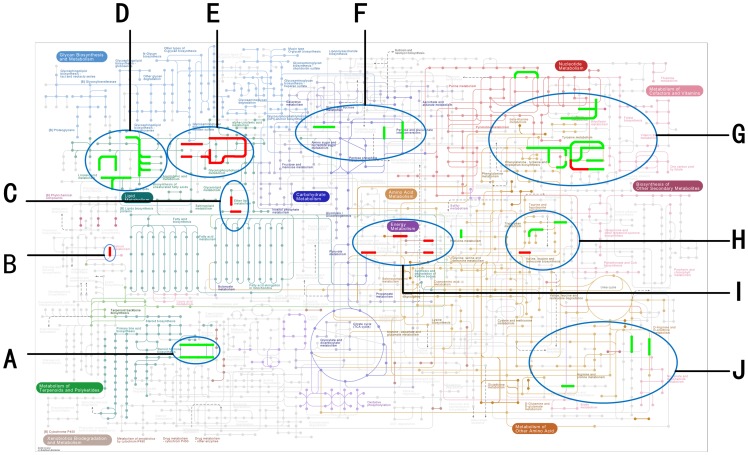
Metabolic networks disturbed by SCI: KEGG mapping. Nodes on the KEGG metabolic pathway for SCI severity (red) and BBB scoring (green) are shown as a series of disturbed metabolic modules in the network. (A) Estrogen and androgen transformation; (B) retinal metabolism; (C) enteral metabolism; (D) glycosphingolipid biosynthesis; (E) glycerophospholipid metabolism; (F) pentose and glucuronate interconvertion, glycoaminoglycan biosynthesis; (G) tyrosine and caffeine metabolism (H) choline to betaine aldehyde, serotonin metabolism (I); ethanol to ethanol, chloroalkene and chloroalkene degradation; and (J) cadaverine and putrescine metabolism.

After metabolites related to severe SCI were identified, metabolite roles and interactions were investigated on the global metabolic network. A three-step method was developed and included the following: 1) HMDB-based identification of enzymes that catalyze metabolites; 2) enzyme ID conversion from HMDB to KEGG enzyme IDs; 3) the use of Matlab KEGG API to map the enzymes into metabolic pathways (KEGG metabolic pathway ID: PATHWAY: map01100). Following this, disturbed metabolic pathways and metabolic pathways related to neurobehavioral recovery after severe SCI were visualized (Figure 5).

Seven metabolic pathways related to severe SCI and eight metabolic pathways related to neurobehavioral recovery were identified (Table 3).

**Table 3:** Metabolic pathways related to severe SCI and neurobehavioral recovery.

Metabolic Pathways Related to Severe SCI and Neurobehavioral Recovery		
	Severe SCI	Neurobehavioral Recovery
**Shared Metabolic Pathways**	1. Glycerophospholipid metabolism2. Etheral metabolism3. Tyrosine metabolism	
**Distinct Metabolic Pathways**	1. Retinal metabolism2. Chloroalkene and chloroalkene degradation3. Choline to betaine aldehyde metabolism	1. Galactose and 4,6-phospho-D-gluconate metabolism2. Cadaverine and putrescine metabolism3. Caffeine metabolism4. Histamine to histidine metabolism5. Taurine and hypotaurine metabolism (Tryptophan metabolism)

Note: Three metabolic pathways are related to both severe SCI and neurobehavioral recovery.

## Discussion

We successfully use plasma 1H-NMR spectra to construct an Eigen-metabolome-based quantitative SCI severity evaluation model, which poses clinical application potential in future. Fifteen metabolites made up an Eigen-metabolome that distinguished severe SCI from mild injuries and healthy controls, whereas sixteen metabolites made up an Eigen-metabolome that distinguished neurobehavioral recovery rats from other treatment groups.

We established a PLS linear formulation group to provide a severe SCI evaluation model. In our model, X represented the ppm value from 1H-NMR spectra and Y represented the severity of SCI. We found that we could distinguish control, severe, and mild injury rats in the PLS score plot by using SVM. To avoid the challenge by excessive clustering and small sample size, we used RMSE to evaluate the accuracy of our model. RMSE of our model (4.76) indicated it was appropriate for evaluating injury severity.

Although few studies demonstrate that SCI disturbs plasma metabolites and metabolic patterns, SCI metabolic disturbances continue to challenge researchers. Consistent with our study, Fujieda Y *et al.* found that neurobehavioral recovery after SCI is related to a decrease in N-acetyl-aspartyl-glutamate and N-acetyl-aspartate in a liquid chromatography–mass spectrometry (LC–MS) metabolomic model [35]. However, this study focused their analysis on a few biomarker(s). To approach comprehensive understanding the complex network after SCI, to identify a few biomarkers is not enough.

We introduced a new approach that integrates metabolomic analysis with metabolic network analysis. Significantly altered metabolites were extracted from the Eigen-metabolome and mapped onto a metabolic network. Enzymes that produce these metabolites were also identified and included in the network. Hundreds of plasma metabolites are altered after major trauma because enzyme activity increases or decreases to effectively decrease or increase metabolites levels [18]. We found that the following metabolic paths were disturbed:

**1) Retinal metabolism (** **Figure 5B** **)**

The retinal metabolism metabolic pathway contains two important metabolites, retinaldehyde (vitamin A) and retinoic acid (RA). RA plays a role in neural differentiation during embryonic differentiation. Studies have shown that RA possesses a neuroprotective effect during central nervous system injury. Plasma vitamin A, vitamin E, and beta-carotene are decreased after SCI. Furthermore, a positive correlation between plasma vitamin A and the severity of the chronic SCI has been identified [22]. A previous study found that RA at the injury site is increased after SCI [23].

**2) Glycerophospholipid and arachidonic acid (AA) metabolism (** **Figure 5D–E** **)**

Phosphoglyceride and sphingomyelin could be used to distinguish the severity of injury in 1H-NMR spectra. Both phospholipids are regulated by superoxide dismutase (SOD) and glutathione (GSH). SCI is currently considered as a myelin sheath damage. The myelin sheath is a greatly extended and modified cell membrane that is wrapped around the nerve axon in a spiral manner. Sphingomyelin and glycerol phospholipid are important components of the cell membrane, and when their metabolic paths are disturbed, the cell membrane is damaged. The simple sphingolipid metabolites ceramide and sphingosine-1-phosphate are major signaling cascade mediators involved in apoptosis, proliferation, stress responses, necrosis, inflammation, autophagy, senescence, and differentiation [19], [20]. Consequently, cell membrane damage releases free fatty acid, particularly AA, from cell membranes. AA leads to secondary spinal cord neuron damage. The results of our study strongly support the hypothesis that free fatty acids contribute to tissue injury following spinal cord trauma [21].

**3) The NAD–NADPH conversion process (** **Figure 5C, E, and I** **)**

The NAD–NADPH conversion process includes etheral metabolism, ethanol–ethylal conversion, and choline to betaine aldehyde metabolism. Many anabolic pathways such as lipid synthesis, cholesterol synthesis, cellular respiration via the electron-transport chain, and fatty acid chain elongation are related to NAD and NADPH. Metabolomic fingerprints change after SCI, partly because of alterations in dehydrogenase activity.

**4) Tyrosine metabolism (** **Figure 5G** **)**

We found that the disturbances in tyrosine metabolism contributed to injury severity and neurobehavioral recovery. Tyrosine metabolism involves various neurotransmitter synthesis pathways. Tyrosine can be converted to L-DOPA, which is converted to the nerve injury biomarkers dopamine, norepinephrine (noradrenaline), and epinephrine (adrenaline). The production and elimination of nitrotyrosine (NT) are markers of nerve injury during tyrosine metabolism. NT is a product of tyrosine nitration mediated by reactive nitrogen species such as the peroxynitrite anion and nitrogen dioxide. NT is one of the most important markers for evaluating tissue and cell damage. Tyrosine nitration leads to enzyme inactivation [22], [23] and affects tyrosine phosphorylation regulation, an important pathway in intracellular signal transduction [24], [25]. Tyrosine nitration also plays a role in apoptosis [26]. Many diseases such as ischemia–reperfusion injury [27], chronic rejection after organ transplantation [28], cardiovascular complications of diabetes [29], and Alzheimer's disease [30] are accompanied by high NT levels, further highlighting the importance of protein tyrosine nitration in tissue damage.

**5) Cadaverine and putrescine metabolism (** **Figure 5J** **)**

The role of decarboxylase ornithine decarboxylase (ODC) as a catalytic product in SCI has gained interest. ODC and putrescine levels significantly increase after SCI [3], [31], [32]. We hypothesize that coordinating these processes and other sequential processes such as spermine expression, enhancement of nitric oxide, and purinergic pathways will be effective to influence innate immune processes after SCI [33].

**6) Disturbances in ether and alcohol metabolism (** **Figure 5C, H, and J** **)**

We found that several metabolic pathways such as chloroalkene and chloroalkene degradation and aldehyde and ethanol transformation (not well studied in SCI) appeared in the disturbance pathways. These metabolites belong to etheral metabolism and are primarily generated by the gut microbiome. This disturbance certainly takes place in clinical practice; however, it is typically ignored in traditional trauma biomedical research.

In summary, SCI can be understood as disturbances in cellular signaling regulatory networks in response to trauma. Furthermore, these disturbances commonly present a pattern of general cellular transduction, as illustrated in Figure 3. It is possible to monitor and assess SCI cellular signaling and modulation changes by profiling the global metabolome and visualizing metabolic and gene paths into distinguishable patterns.

Our study demonstrated that 1H-NMR spectra can be used to quantitatively measure and visualize the metabolic network disturbances after SCI. We identified major metabolites and enzymes through unbiased screening of compounds identified by metabolomic analysis. Metabolites were mapped to a metabolic network, and their functions were interpreted by KEGG metabolic network visualization. This method has the advantage of integrating metabolic research with the trauma mechanisms to aid our understanding of the pathophysiological SCI process.

Our model incorporated metabolomics, biological informatics, and pattern recognition to develop a new evaluation and outcome prediction tool for SCI that can provide substantial improvements to the diagnosis and intervention monitoring of SCI patients.

## Supporting Information

Table S1 **The Basso, Beattie, and Bresnahan (BBB) score scale of all rats.**
(XLSX)

Table S2 **Raw data of 1H-NMR: All the raw data through binning process of the NMR spectrum after Fourier transformation as a 200×29 matrix; Each row describes the integral binning value of a spectrum for a sample.**
(XLSX)

Table S3 **Grouping of animals: The treatment that 29 samples received are as follows: “control” present, control group; “mild” present, the rat received a laminectomy without hemisection of spinal cord injury; “op” present, the rat received a hemisection of the spinal cord and was scarified 3 days after the operation; “op7” present, the rat received a hemisection of the spinal cord and was scarified 7 days after the operation.**
(XLSX)

## References

[1] Sekhon LHS, Fehlings MG (2001) Epidemiology, demographics, and pathophysiology of acute spinal cord injury. Spine 26: S2–S12. 11805601 10.1097/00007632-200112151-00002

[2] Li JJ, Zhou HJ, Hong Y (2004) Spinal cord injuries in Beijing: a municipal epidemiological survey in 2002. Chinese Journal of Rehabilitation Theory and Practice 10: 412–413 (in Chinese).

[3] Long YC, Kostovski E, Boon H, Hjeltnes N, Krook A, et al (2011) Differential expression of metabolic genes essential for glucose and lipid metabolism in skeletal muscle from spinal cord injured subjects. J Appl Physiol 110: 1204–1210. 21393466 10.1152/japplphysiol.00686.2010

[4] Varma AK, Das A, Wallace IV, Barry J, Vertegel AA, et al (2013) Spinal cord injury: a review of current therapy, future treatments, and basic science frontiers. Neurochem Res 38: 895–905. 23462880 10.1007/s11064-013-0991-6 PMC4103794

[5] Biering-Sørensen B, Kristensen IB, Kjaer M, Biering-Sørensen F (2009) Muscle after spinal cord injury. Muscle Nerve 40: 499–519. 19705475 10.1002/mus.21391

[6] Duckworth W, Jallepalli P, Solomon S (1983) Glucose intolerance in spinal cord injury. Arch Phys Med Rehabil 64: 107. 6338859

[7] Jeon JY, Weiss CB, Steadward RD, Ryan E, Burnham RS, et al (2002) Improved glucose tolerance and insulin sensitivity after electrical stimulation-assisted cycling in people with spinal cord injury. Spinal Cord 40: 110–117. 11859437 10.1038/sj.sc.3101260

[8] de Groot S, Dallmeijer AJ, Post MW, Angenot EL, van der Woude LH (2008) The longitudinal relationship between lipid profile and physical capacity in persons with a recent spinal cord injury. Spinal Cord 46: 344–351. 18026171 10.1038/sj.sc.3102147

[9] Huang TS, Wang YH, Lee SH, Lai JS (1998) Impaired hypothalamus-pituitary-adrenal axis in men with spinal cord injuries. Am J Phys Med Rehabil 77: 108–112. 9558010

[10] Clark MJ, Schopp LH, Mazurek MO, Zaniletti I, Lammy AB, et al (2008) Testosterone levels among men with spinal cord injury: relationship between time since injury and laboratory values. Am J Phys Med Rehabil 87: 758–767. 18716488 10.1097/PHM.0b013e3181837f4f

[11] Tsitouras PD, Zhong YG, Spungen AM, Bauman WA (1995) Serum testosterone and growth hormone/insulin-like growth factor-I in adults with spinal cord injury. Horm Metab Res 27: 287–292. 7557841 10.1055/s-2007-979961

[12] Bugaresti JM, Tator CH, Silverberg JD, Szalai JP, Malkin DG, et al (1992) Changes in thyroid hormones, thyroid stimulating hormone and cortisol in acute spinal cord injury. Paraplegia 30: 401–409. 1635789 10.1038/sc.1992.90

[13] Nemunaitis GA, Mejia M, Nagy JA, Johnson T, Chae J, et al (2010) A descriptive study on vitamin D levels in individuals with spinal cord injury in an acute inpatient rehabilitation setting. PM R 2: 202–208. 20359685 10.1016/j.pmrj.2010.01.010

[14] LeBrasseur NK, Walsh K, Arany Z (2011) Metabolic benefits of resistance training and fast glycolytic skeletal muscle. Am J Physiol Endocrinol Metab 300: E3–E10. 21045171 10.1152/ajpendo.00512.2010 PMC3023213

[15] Zhang GF, Sadhukhan S, Tochtrop GP, Brunengraber H (2011) Metabolomics, pathway regulation, and pathway discovery. J Biol Chem 286: 23631. 21566142 10.1074/jbc.R110.171405 PMC3129142

[16] Wold S, Sjostrom M, Eriksson L (2001) PLS-regression: a basic tool of chemometrics. Chemometr Intell Lab 58: 109–130.

[17] Jiang H, Peng J, Zhou ZY, Duan Y, Chen W, et al (2010) Establishing 1H nuclear magnetic resonance based metabonomics fingerprinting profile for spinal cord injury: a pilot study. Chin Med J 123: 2315–2319. 21034541

[18] Alves R, Chaleil RAG, Sternberg MJE (2002) Evolution of enzymes in metabolism: a network perspective. J Mol Bio 320: 751–770. 12095253 10.1016/s0022-2836(02)00546-6

[19] Hannun YA, Obeid LM (2002) The Ceramide-centric universe of lipid-mediated cell regulation: stress encounters of the lipid kind. J Biol Chem 277: 25847–25850. 12011103 10.1074/jbc.R200008200

[20] Spiegel S, Milstien S (2002) Sphingosine 1-phosphate, a key cell signaling molecule. J Biol Chem 277: 25851–25854. 12011102 10.1074/jbc.R200007200

[21] Toborek M, Malecki A, Garrido R, Mattson MP, Hennig B, et al (1999) Arachidonic acid-induced oxidative injury to cultured spinal cord neurons. J Neurochem 73: 684–692. 10428065 10.1046/j.1471-4159.1999.0730684.x

[22] Radi R (2004) Nitric oxide, oxidants, and protein tyrosine nitration. Proc Natl Acad Sci U S A 101: 4003–4008. 15020765 10.1073/pnas.0307446101 PMC384685

[23] Ara J, Przedborski S, Naini AB (1998) Inactivation of tyrosine hydroxylase by nitration following exposure to peroxynitrite and 1-methyl-4-phenyl-1, 2, 3, 6-tetrahydropyridine (MPTP). Proc Natl Acad Sci U S A 95: 7659–7663. 9636206 10.1073/pnas.95.13.7659 PMC22714

[24] Wang YT, Salter MW (1994) Regulation of NMDA receptors by tyrosine kinases and phosphatases. Nature 369: 233–235. 7514272 10.1038/369233a0

[25] García-Cardeña G, Fan R, Stern DF (1996) Endothelial nitric oxide synthase is regulated by tyrosine phosphorylation and interacts with caveolin-1. J Biol Chem 271: 27237–27240. 8910295 10.1074/jbc.271.44.27237

[26] Mattson MP, Goodman Y, Luo H (1997) Activation of NF-κctivation of NF ical Chemistry, is regulated by tyrosine phosphorylation and interacts with caveolin-1. l-1, 2, 3, 6-tetrahydropyridine (MPTP) the network. (ionast sqoduction and protein tyrosine nitration. J Neuro Res 49: 681–697. 10.1002/(SICI)1097-4547(19970915)49:6<681::AID-JNR3>3.0.CO;2-3 9335256

[27] Liu B, Tewari AK, Zhang L (2009) Proteomic analysis of protein tyrosine nitration after ischemia reperfusion injury: mitochondria as the major target. Biochim Biophys Acta 1794: 476–485. 19150419 10.1016/j.bbapap.2008.12.008 PMC2637933

[28] MacMillan-Crow LA, Crow JP, Kerby JD (1996) Nitration and inactivation of manganese superoxide dismutase in chronic rejection of human renal allografts. PNAS 93: 11853–11858. 8876227 10.1073/pnas.93.21.11853 PMC38148

[29] Turko IV, Li L, Aulak KS (2003) Protein tyrosine nitration in the mitochondria from diabetic mouse heart Implications to dysfunctional mitochondria in diabetes. J Biol Chem 278: 33972–33977. 12821649 10.1074/jbc.M303734200

[30] Good PF, Werner P, Hsu A (1996) Evidence of neuronal oxidative damage in Alzheimer's disease. Am J Path 149: 21. 8686745 PMC1865248

[31] Mautes AE, Paschen W, Röhn G, Nacimiento AC (1999) Changes in ornithine decarboxylase activity and putrescine concentrations after spinal cord compression injury in the rat. Neurosci Lett 264: 153–156. 10320037 10.1016/s0304-3940(99)00197-4

[32] Gonzalez S, Coirini H, Gonzalez Deniselle MC, Gonzalez S, Calandra R, et al (1995) Time-dependent effects of dexamethasone on glutamate binding, ornithine decarboxylase activity and polyamine levels in the transected spinal cord. J Steroid Biochem Mol Biol 55: 85–92. 7577724 10.1016/0960-0760(95)00160-2

[33] Dibaj P, Nadrigny F, Steffens H, Scheller A, Hirrlinger J, et al (2010) NO mediates microglial response to acute spinal cord injury under ATP control in vivo. Glia 58: 1133–1144. 20468054 10.1002/glia.20993

[34] Kinross JM, Alkhamesi N, Barton RH, Silk DB, Yap IK, et al (2011) Global metabolic phenotyping in an experimental laparotomy model of surgical trauma. J Proteome Res 10: 277–287. 21105667 10.1021/pr1003278

[35] Fujieda Y, Ueno S, Ogino R, Kuroda M, Jönsson TJ, et al (2012) Metabolite profiles correlate closely with neurobehavioral function in experimental spinal cord injury in rats. PLOS ONE 7: e43152. 22912814 10.1371/journal.pone.0043152 PMC3418274

